# Visible to near-IR fluorescence from single-digit detonation nanodiamonds: excitation wavelength and pH dependence

**DOI:** 10.1038/s41598-018-20905-0

**Published:** 2018-02-06

**Authors:** Philipp Reineck, Desmond W. M. Lau, Emma R. Wilson, Nicholas Nunn, Olga A. Shenderova, Brant C. Gibson

**Affiliations:** 1 ARC Centre of Excellence for Nanoscale BioPhotonics & School of Science, RMIT University, Melbourne, VIC 3001 Australia; 2 Adámas Nanotechnologies, Inc., 8100 Brownleigh Drive, Suite 120, Raleigh, North Carolina 27617 United States

## Abstract

Detonation nanodiamonds are of vital significance to many areas of science and technology. However, their fluorescence properties have rarely been explored for applications and remain poorly understood. We demonstrate significant fluorescence from the visible to near-infrared spectral regions from deaggregated, single-digit detonation nanodiamonds dispersed in water produced via post-synthesis oxidation. The excitation wavelength dependence of this fluorescence is analyzed in the spectral region from 400 nm to 700 nm as well as the particles’ absorption characteristics. We report a strong pH dependence of the fluorescence and compare our results to the pH dependent fluorescence of aromatic hydrocarbons. Our results significantly contribute to the current understanding of the fluorescence of carbon-based nanomaterials in general and detonation nanodiamonds in particular.

## Introduction

Detonation nanodiamonds (DNDs) are of great significance to many areas of science and engineering today. This is mainly due to their exceptional chemical stability, tunable surface chemistry, economical synthesis, small primary particle size of 4–6 nm and high biocompatibility^1,2^. Relative to other properties of DND particles, their fluorescence properties have received far less attention. Understanding the precise relationship between chemical structure and optoelectronic properties of materials such as carbon dots, graphene oxide and detonation nanodiamonds remains a major challenge in the area of carbon-based fluorescent nanomaterials - not least because of the lack of a chemically and physically well-defined model system.

In general, it is crucial to clearly distinguish different types of nanodiamonds. For example the physical and chemical properties of high-pressure high-temperature nanodiamonds are profoundly different from those of DNDs^3^. Purification protocols, surface chemistry, particle size and aggregation state are equally important and critically influence particle characteristics in general^4^ and fluorescence properties in particular^5^. This study focuses on the fluorescence properties of deaggregated and oxidised DNDs, which are also to be distinguished from DND clusters of several tens or hundreds of nanometers in size.

The fluorescence of aggregated DND particles has been investigated in several publications^6–9^, however most of these use irradiated and annealed particles^10–12^. Far fewer reports on the fluorescence of deaggregated DNDs exist^13,14^. Recently, fluorescence from deaggregated DNDs dispersed in water and water-ethanol solutions has been reported^13^, as well as a ‘red edge’ effect in fluorescence spectra of water suspensions of 10 nm DNDs^15^. More commonly, DNDs are investigated in a dry state on a glass or silicon substrate. Here, both fluorescence from nitrogen-vacancy color centers has been observed in a small subset of isolated 5 nm DND particles^16^ as well as a broad, featureless fluorescence that is not photostable^8,11^. The latter has generally been attributed to non-diamond carbon, while the exact photophysical origin of this fluorescence remains unknown^7,10,12^.

In this study, we report the excitation wavelength dependent fluorescence from the visible to the near-infrared spectral region of deaggregated DNDs dispersed in water. Absorption and scattering properties of DNDs are determined and their excitation wavelength dependent fluorescence is investigated for the spectral range from 400 nm to 700 nm. The pH dependence of this fluorescence is also investigated for the first time. We show that the pH dependence is remarkably similar to that of several aromatic hydrocarbons. We expect our results to encourage even more research into the origins of DND fluorescence and open up the possibility of employing DND NPs as a pH sensors and multifunctional biomarkers.

## Results

### Material synthesis and characterization

Detonation nanodiamonds investigated in this paper were produced by oxidation of a detonation soot using graphite intercalating acids (mixture of nitric/sulfuric acids) that have previously been reported to create nanodiamonds with significantly enhanced fluorescence as compared to the DND particles obtained by oxidation of detonation soot by other means such as oxidation in air^9^. It was hypothesized that this enhanced fluorescence originates from carbon dots observed on the nanoparticles surface using high-resolution transmission electron microscopy (HRTEM) images. The particles investigated here have a partially carboxylated surface^13^ (see SI Figure S10 for Fourier-transform infrared (FTIR) spectra) and are well dispersed in water. Suspensions of 1 mg mL^−1^ were investigated in all experiments using a custom-built setup fluorescence spectroscopy setup. For pH dependent measurements the pH was adjusted using HCl and NaOH. See Methods and Supplementary Information (SI) for details on material processing and sample preparation.

Dispersed in water at neutral pH, the particles are colloidally stable with a zeta potential of about −60 mV and a particle size of 5 nm as determined by dynamic light scattering (DLS). See SI Figure S2 for DLS size distributions. Energy-dispersive X-ray spectroscopy (EDS) shows that the particles are composed of carbon, but also contain significant amounts of oxygen. Electron energy loss spectroscopy (EELS) experiments demonstrate that carbon is mainly present in the form of diamond (82% sp^3^ hybridized carbon) and only to 18% of sp^2^ bonded carbon (See SI Figure S3 and S4 for EDS and EELS results, respectively). In agreement with previous reports^9^, HRTEM images of the particles show both highly regular lattice structures of crystalline diamond as well as less ordered forms of carbon surrounding the diamond cores (see SI Figure S9). The partial carboxylation of the surface was verified using Fourier-transform infrared spectroscopy (FTIR, see SI Figure S10); the spectrum shows absorption peaks characteristic of O-H bend (~1640 cm^−1^) and C=O stretch vibrations (~1750 cm^−1^) in carboxylate groups.

### Extinction and fluorescence properties

**Figure 1:**
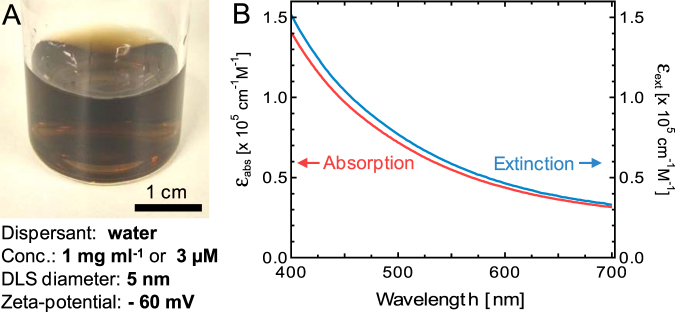
(**A**) Image of an aqueous DND solution and summary of important properties. (**B**) Molar absorption and extinction coefficient of DND particles in water as a function of wavelength. See main text and Supplemental Information for details.

The NP solution shows a brown color (Fig. 1A) caused by light absorption. An extinction spectrum was acquired as well as an absorption spectrum using an integrating sphere. The resulting estimated molar absorption (ε_abs_) and extinction coefficients (ε_ext_) are shown in Fig. 1B for the spectral region from 400 nm to 700 nm. (See Experimental Methods and SI for details). The light absorption decreases monotonically towards longer wavelength by about 5 times in this spectral range. At 400 nm only about 7% of the extinction (= scattering + absorption) is caused by scattering - presumably caused by some of the larger particle aggregates in solution. The magnitude of ε_abs_ of 1 × 10^5^ cm^−1^ M^−1^ is noteworthy considering that the particles mostly consist of diamond (as opposed to graphitic or amorphous carbon), which in its pure form is a highly transparent material throughout the visible and near-infrared spectral region. It is also on the same order of magnitude as the absorption coefficient of carbon dots^17^.

**Figure 2:**
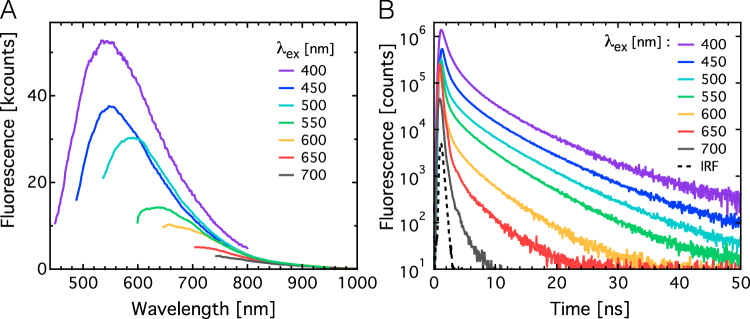
Excitation wavelength dependence of fluorescence spectra (**A**) and time-resolved fluorescence decay traces (**B**) of DND particles dispersed in water. Samples were excited in the spectral range from 400 nm to 700 nm as indicated in the graphs. The instrument response function (IRF, panel B, black dashed line) is also shown.

Fluorescence spectra of the DND NPs dispersed in deionized (DI) water are shown in Fig. 2A for excitation wavelengths (λ_ex_) between 400 nm and 700 nm. All spectra were corrected for contributions from the water Raman signal, which was at least an order of magnitude weaker than the fluorescence signal from DND NPs (see SI Figure S5). Fluorescence is most efficiently excited at 400 nm and the fluorescence intensity decreases continuously to λ_ex_ of 700 nm. Time-resolved fluorescence traces show two main decay components: a fast component below 1 ns and a slower decay above 1 ns (Fig. 2B). With increasing excitation wavelength, the fast decay component becomes more dominant and the overall decay at λ_ex_ = 700 nm approaches the instrument response function (IRF) of the system.

**Figure 3:**
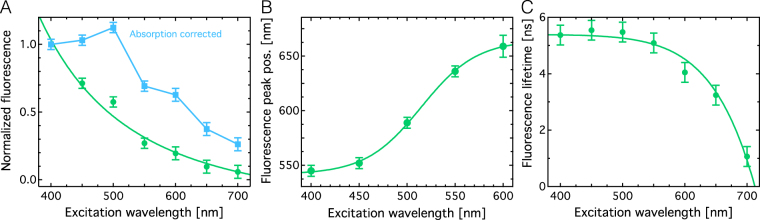
Analysis of the excitation wavelength dependence of DND fluorescence. (**A**) Normalized fluorescence intensity inferred from Fig. 2A directly (green markers) and normalized fluorescence corrected for differences in absorption shown in Fig. 1A (blue markers). (**B**) Fluorescence emission peak position. (**C**) Fluorescence lifetime τ_2_ of the slow decay component. All lines are a guide to the eye only. See SI for details on data analysis and fitting.

The overall fluorescence intensity shows a monotonic decrease for increasing λ_ex_ (Fig. 3A, green line). The fluorescence at λ_ex_ = 700 nm is about 16 times weaker than for λ_ex_ = 400 nm excitation. The data was also corrected for differences in absorption (Fig. 1B) and is shown for comparison in Fig. 3A (yellow line). The absorption corrected trace shows a maximum at λ_ex_ = 500 nm and a much weaker overall excitation wavelength dependence with the fluorescence at λ_ex_ = 400 nm only 4 times stronger than at λ_ex_ = 700 nm.

In the same excitation wavelength region, the spectral fluorescence peak position (λ_em_) red-shifts by about 100 nm from about 550 nm (λ_ex_ = 400 nm) to about 650 nm for λ_ex_ = 700 nm (Fig. 3B). This means a reduction in Stokes-shift from 150 nm to only 50 nm. The longer fluorescence lifetime (slower decay) component remains largely constant between λ_ex_ of 400 nm and 550 nm and then decreases rapidly towards longer wavelengths (Fig. 3C).

The fluorescence brightness B is generally defined as the product of fluorescence quantum yield (Φ) and the absorption coefficient (ε_abs_). Figure 1B shows that the absorption of light decreases with increasing excitation wavelength. Compensating for this effect reveals relative changes in the quantum yield Φ. Therefore, Fig. 3A suggests that Φ is maximal for λ_ex_ of 500 nm. The red-shift of λ_em_ with increasing λ_ex_ suggests that optical transitions of lower energy are excited as the excitation photon energy decreases. A decrease in fluorescence (Fig. 3A) intensity in combination with a decrease in fluorescence lifetime (Fig. 3C) suggest that an increase in non-radiative decay rate mainly causes both effects at higher λ_ex_ rather than a change in radiative decay rate.

For an excitation wavelength of 450 nm, we have determined the fluorescence quantum yield for the DND particles in water to be *Φ*_*DND*_ = 0.22% using fluorescein as a reference (see SI Figure S12 for details). For a molar absorption coefficient of 9.7 × 10^4^ M^−1^cm^−1^ (see Fig. 1B) we estimate the absolute fluorescence brightness to be *B*_*DND*_ = 2.1 × 10^2^ M^−1^cm^−1^. Compared to fluorescein (*B*_*F*_ = 7.6 × 10^4^ M^−1^cm^−1^, using values reported by Kubista *et al*.^18^), which is one of the brightest fluorescent molecules known, the fluorescence from the DND particles investigated here is more than two orders of magnitude less bright. This difference in brightness is mainly the result of a difference in quantum yield, while the absorption coefficients are on the same order of magnitude.

### pH dependent fluorescence

**Figure 4:**
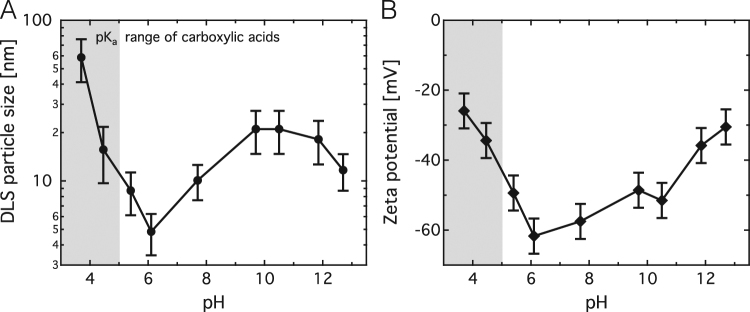
DND particle size and zeta potential as a function of pH. (**A**) Particle diameter determined via dynamic light scattering. (**B**) Zeta potential. Aqueous solutions of HCl and NaOH were used to adjust the pH. Light grey regions indicate the pK_a_ range of carboxylic acids.

To investigate the role of the particle surface in the fluorescence process, the pH of the DND solutions was varied between pH 3.7 and 12.7 using HCl and NaOH, respectively. We find the particles to show the smallest average particle diameter of about 5 nm and highest zeta potential of about − 60 mV at close to neutral pH in DI water without the addition of HCl or NaOH (Fig. 4A and B). At this pH, carboxylic acid surface groups are mostly deprotonated (COO^−^)^19^ and lead to high colloidal stability through electrostatic repulsion. A reduction in pH causes the stepwise protonation of these groups, leading to an increase in zeta potential (Fig. 4B) and induces particle aggregation (Fig. 4A). The pK_a_ of carboxylic acids strongly depends on the molecule or particle it is bound to^19^ and is therefore indicated as a region rather than a specific point in Fig. 4.

Interestingly, an increase in pH also leads to an increase in particle size (partial aggregation) as well as a slight increase in zeta potential at pHs between 8 and 10, and a strong increase above pH 11. This aggregation and increase in zeta potential at basic pHs is most likely caused by the shielding of negative particle surface charges by Na^+^ ions. At pH 12 for example the concentration of Na^+^ ions in solution is 5 mM. This reduces the electrostatic repulsion between particles and therefore the Debye shielding length by two orders of magnitude relative to deionized water, which considerably reduces the absolute value of the zeta potential and hence the colloidal stability of the particles. (See SI for calculation of the Debye length).

At pHs <5 we find particles aggregate and flocculate on a timescale of 30 minutes. All other particle solutions at pH > 5 show no sign of flocculation over days. Therefore, HCl and NaOH were added to the DND solutions immediately before experiments and experiments conducted within less than 1 minute after the addition.

**Figure 5:**
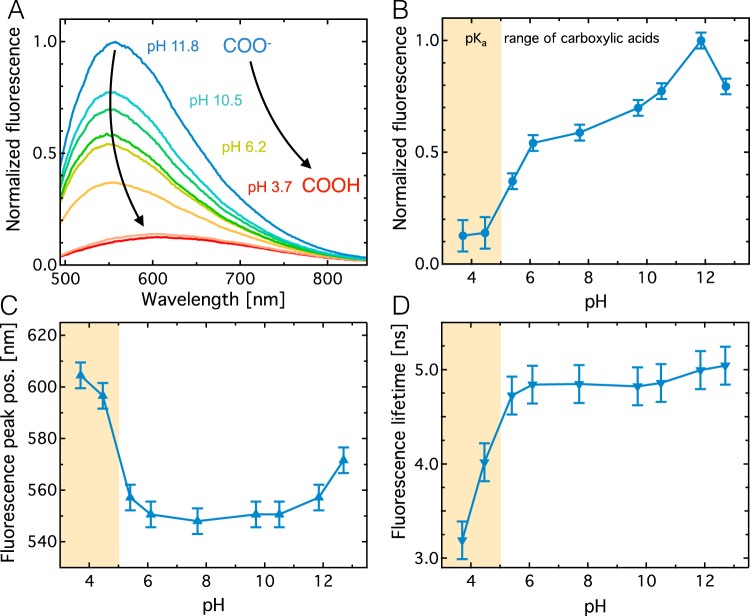
pH dependence of the fluorescence of DND particles in water. (**A**) Fluorescence spectra for NP solutions of pH 11.8 to pH 3.7. Excitation wavelength is 450 nm in all cases. (**B**) Normalized fluorescence intensity as a function of pH. (**C**) Spectral fluorescence peak position. (**D**) Long fluorescence lifetime component (τ_2_). See SI Figure S6 and S7 for fluorescence decay raw data and analysis. Yellow regions indicate the pK_a_ range of carboxylic acids.

DND fluorescence spectra for the pH region between 11.8 and 3.7 are shown in Fig. 5A (λ_ex_ = 450 nm). From close to neutral pH of 6.2, the fluorescence intensity sharply decreases towards lower pH levels, and increases with increasing pH and peaks at a pH of 11.8 (Fig. 5B). At basic pHs both λ_em_ and τ_2_ only show a very weak pH dependence and remain largely constant (Fig. 5C and D). At acidic pHs, λ_ex_ increases sharply between pH 5.4 and 4.5, which coincides with a decrease in fluorescence lifetime τ_2_. The fact that the fluorescence intensity increases at basic pHs while the lifetime remains constant demonstrates that a decrease in the non-radiative decay rate (k_nr_) causes the intensity increase. Similarly, the shortening of the lifetime in the acidic pH region suggests that an increase in k_nr_ results in the observed intensity decrease.

These changes are *not* caused by aggregation. We find that the decrease in fluorescence upon addition of HCl occurs in less than 500 ms (see SI Figure S8). The mean square displacement during 500 ms of diffusion of a 5 nm particle in 3 dimensions in water is well below 1 nm. This makes a collision with another particle within this timeframe highly improbable and excludes aggregation as a possible cause for the observed decrease even though it does occur on the timescale of hours (see SI for calculations). Furthermore, partial particle aggregation also occurs in the basic pH region (see Fig. 4A), where we observe an *increase* in fluorescence.

In addition to that we have performed experiments using NaCl instead of HCl to induce aggregation without changing the pH. The results are shown in SI Figure S8 and demonstrate that the addition of 250 µM NaCl does not change the DND fluorescence significantly (<1%), while the same concentration of HCl causes a decrease in fluorescence of more than 70%.

## Discussion

The fluorescence of oxidized detonation nanodiamonds has been investigated in aqueous and water-ethanol mixtures solution by Vervald and co-workers^13^. It is important to note that the partial carboxylation of the DNDs used in this study was obtained via oxidation in air at 420 °C and not via oxidation in sulfuric and nitric acid as reported in our study (see Methods Section for details). While the shape and spectral position of the fluorescence spectra reported in this study for an excitation wavelength of 405 nm is similar to the spectra reported here, the intensity is more than an order of magnitude lower relative to the water Raman signal. Dolenko *et al*. have also reported a red-shift of DND fluorescence for excitation wavelengths between 405 nm and 532 nm for air oxidized detonation nanodiamonds^15^ - again with significantly lower fluorescence than seen in our experiments.

To the best of our knowledge, the pH dependence of fluorescence spectra and fluorescence lifetime and excitation wavelength dependence of fluorescence lifetime has not been reported for deaggregated, single-digit detonation nanodiamonds. The fluorescence of larger, *not* fully deaggregated DND particles dispersed in water has also been investigated in several publications^6,20^. These results all differ significantly from our findings, suggesting that chemically different materials were investigated. Chung *et al*^14^. report fluorescence spectra from deaggregated DND dried (which generally leads to aggregation) on a silicon substrate for 488 nm and 532 nm excitation and also find a red-shift in λ_em_ for longer excitation wavelengths. The absorption spectrum and the excitation wavelength dependence of deaggregated DND particles in water we report here are strikingly similar to those observed for many different types of carbon dots. However, these generally consist of sp^2^ bonded (graphitic or amorphous) carbon and several percent of other elements such as N and O, whereas 82% of the DND material investigated here is sp^3^ hybridized carbon (diamond). It is feasible that the remaining 18% of sp^2^ carbon in combination with oxygen in our samples are the main source of fluorescence in analogy to carbon dots. However, this does not explain the fundamental photophysics causing the observed fluorescence, which is still not fully understood for carbon dots.

Fluorescence from several types of carbon dots is known to depend on pH^21,22^. However, the fluorescence intensity has been reported to increase as well as decrease as a function of pH depending on the exact chemical composition of the material^21,22^.

Many aromatic hydrocarbons (AHs) show excitation independent fluorescence in the UV and blue spectral region. Fu *et al*^23^. have reported that the stacking of AHs leads to excitation wavelength dependent fluorescence throughout the visible analogous to that of carbon dots and similar to the spectra shown in Fig. 2A. Interestingly, several water-soluble aromatic hydrocarbons containing COOH groups show a pH dependence very similar to the pH dependence we report for DNDs^19,24,25^. The simplest of those is salicylic acid (or 2-hydroxybenzoic acid), which has a pK_a_ of close to 3^19^. In its deprotonated form at neutral pH it shows a quantum yield of about 0.36, a fluorescence lifetime of about 4 ns and λ_em_ of 408 nm^19^. Upon protonation, the fluorescence is quenched to < 0.01, the lifetime reduced to 0.1 ns and the emission peak red-shifts to 450 nm^19^. All of these observations are in good qualitative agreement with our observations. Similar pH dependent fluorescence has also been reported for larger AHs such as 1-hydroxy-2-naphthoic acid^24^ and 1-pyrenecarboxylic acid^25^. The pH dependent fluorescence of these molecules is rationalized through the concept of excited-state proton transfer^19,25^. Investigating whether a similar process causes the pH dependent fluorescence observed here will be the focus of future studies. To do this, the exact chemical structure in terms of the surface groups of DND NPs, their relative distance, and the pK_a_ of the COOH groups on the DND surface must be determined, which is beyond the scope of this letter. However, our results suggest that simple PAHs, which mainly consist of sp^2^ hybridized carbon, could be the key to understanding the photophysics underlying DND NP fluorescence.

For bioimaging applications, the colloidal stability of particles in high-salt environments such as buffers and cell media is of great importance. It has been demonstrated that DNDs aggregate at relatively low ionic strength electrolytes below 10 mM concentration^26^. However, suitable surface modifications can stabilize DNDs in physiological buffers, making them highly valuable for cellular imaging (unpublished results from our team).

We have shown that deaggregated, COOH functionalized DND particles show excitation wavelength dependent fluorescence. Light absorption, fluorescence quantum yield and fluorescence lifetime all decrease with increasing excitation wavelength, which coincides with a red-shift in fluorescence of about 100 nm. DND fluorescence is most efficiently excited at 400 nm in the investigated spectral region. These characteristics are analogous to those commonly observed for carbon dots. The highest fluorescence is found at a pH of 11.8, which decreases by almost one order of magnitude as the pH is reduced to 3.7. This change coincides with a successive protonation of COO^−^ groups as the pH is lowered from 11.8 (COO^−^) to 3.7 (mostly COOH). We show that the fluorescence of simple AHs show a qualitatively similar pH dependence as the one observed for DND particles. From pH 6 to 12 the DND particles show a relatively long fluorescence lifetime of close to 5 ns, making fluorescence lifetime imaging (FLIM) for bioimaging applications feasible.

## Methods

Detonation soot was produced via detonation of an oxygen-deficient explosives mixture of trinitrotoluene with hexogen (50:50 wt%) in a closed steel chamber using CO_2_ cooling media. The detonation soot product is a mixture of up to 30 wt% of diamond particulates with other carbon allotropes, as well as metallic impurities, and was subsequently purified by oxidation of the soot in a 1:3 mixture of nitric and sulfuric acid in the presence of sulfur oleate at high temperature (above 200 °C) at the vendor site (FGUP Altay, Russian Federation)^27^. The residual content of incombustible metallic impurities in DNDs was estimated to be 1 wt%. Subsequent purification at Adamas Nanotechnologies using hydrochloric acid (HCl) reduced the metal content to 0.4 wt%. The purified raw diamond aggregates were suspended in deionized water (DI water) and processed in a planetary bead mill (Retsch GmbH) using 300 µm zirconia beads for 2 hours. The milled material was then fractionated using centrifugation at 25,000 RCF for 2 hours to isolate the 5 nm primary particles. For more details on sample preparation see SI.

A pulsed white light laser (WhiteLase WL-SC400, Fianium) was used as an excitation source in a custom built fluorescence spectroscopy setup (see SI Figure S1 for details). Fluorescence was either collected with a spectrometer (SpectraPro, Princeton Instruments fitted with a PIXIS CCD camera) or detected with avalanche photodiodes (SPCM-AQRH-14, Excelitas) and analyzed with a correlator card (Picoquant, TimeHarp 260) for time-resolved measurements.

DLS and zeta potential measurements were obtained with a Zetasizer Nano ZS (Malvern Instruments). Absorption and extinction spectra were acquired with a Cary7000 (Agilent Technologies) fitted with an integrating sphere. See Supporting Information for details on data analysis and fitting.

## Electronic supplementary material

Supplementary Information
