# Supplementary material for: Visible to near-IR fluorescence from single-digit detonation nanodiamonds: excitation wavelength and pH dependence
Source: Sci Rep. 2018 Feb 6;8:2478. doi: 10.1038/s41598-018-20905-0 (PMC5802785; doi:10.1038/s41598-018-20905-0)
Supplement: Supplementary file 1 — Supplementary Information [file 41598_2018_20905_MOESM1_ESM.pdf]

Supporting Information for

Visible to near-IR fluorescence from single-digit detonation nanodiamonds: excitation wavelength and pH dependence

Philipp Reineck[1], Desmond W.M. Lau[1], Emma R. Wilson[1], Nicholas Nunn[2], Olga A. Shenderova[2], Brant C. Gibson[1]

[1]ARC Centre of Excellence for Nanoscale BioPhotonics & School of Science, RMIT University, Melbourne, VIC 3001, Australia.

[2] Adámas Nanotechnologies, Inc., 8100 Brownleigh Drive, Suite 120, Raleigh, North Carolina 27617, United States

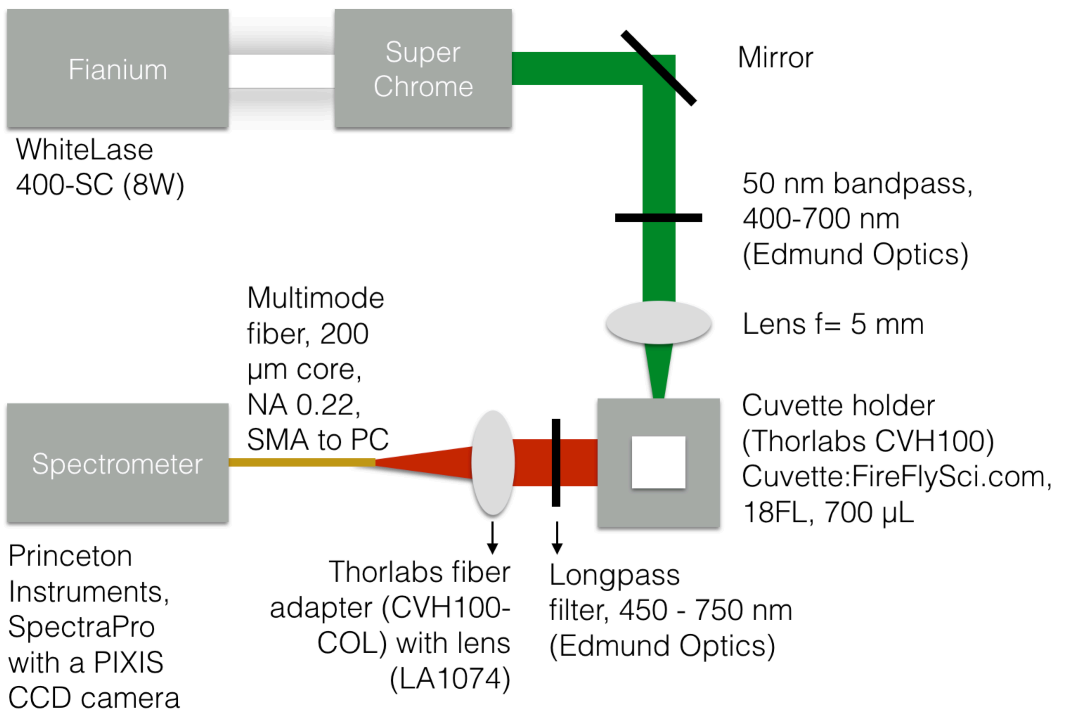

Figure S1. Schematic drawing of the custom built in-solution fluorescence spectroscopy setup.

Experimental parameters used for data acquisition using the above setup

Pulse repetition rate: 80 MHz (fluorescence spectra) / 10 MHz (fluorescence decay)
Spectrometer/ CCD camera: 5 pixel binning, 10 seconds integration time

**Excitation wavelength [nm]**	**50 nm bandpass filter [nm]**	**Longpass filter [nm]**	**Ex intensity [mW]**	**Spectral width FWHM [nm]**
400	400	425	1.24	30
450	450	475	8.55	30
500	500	525	20.6	30
550	550	600	22.2	20
600	600	625	21.9	20
650	650	675	22.4	20
700	700	725	31.1	20

Sample preparation for pH dependent measurements

Sample	NP sol @ 2mg/ml [μL]	HCl @ 1 mM [μL]	NaOH @ 10 mM [μL]	water	Final HCl conc. [μM]	Final NaOH conc. [μM]	pH measured
**1**	200	200	0	0	500	0	3.7
**2**	200	100	0	100	250	0	4.5
**3**	200	50	0	150	125	0	5.4
**4**	200		0	200	0	0	6.1
**5**	200	0	2	198	0	50	7.7
**6**	200	0	10	190	0	250	9.7
**7**	200	0	20	180	0	500	10.5
**8**	200	0	200	0	0	5000	11.8
**9**	200	0	200 (100 mM)	0	0	50000	12.7

Table S1. Nanoparticle (NP) solution, HCl and NaOH solutions used to prepare the samples investigated in this study. Sample 4 (no HCl or NaOH) was used to investigate the excitation wavelength dependence shown in Figure 2 and 3 in the main text.

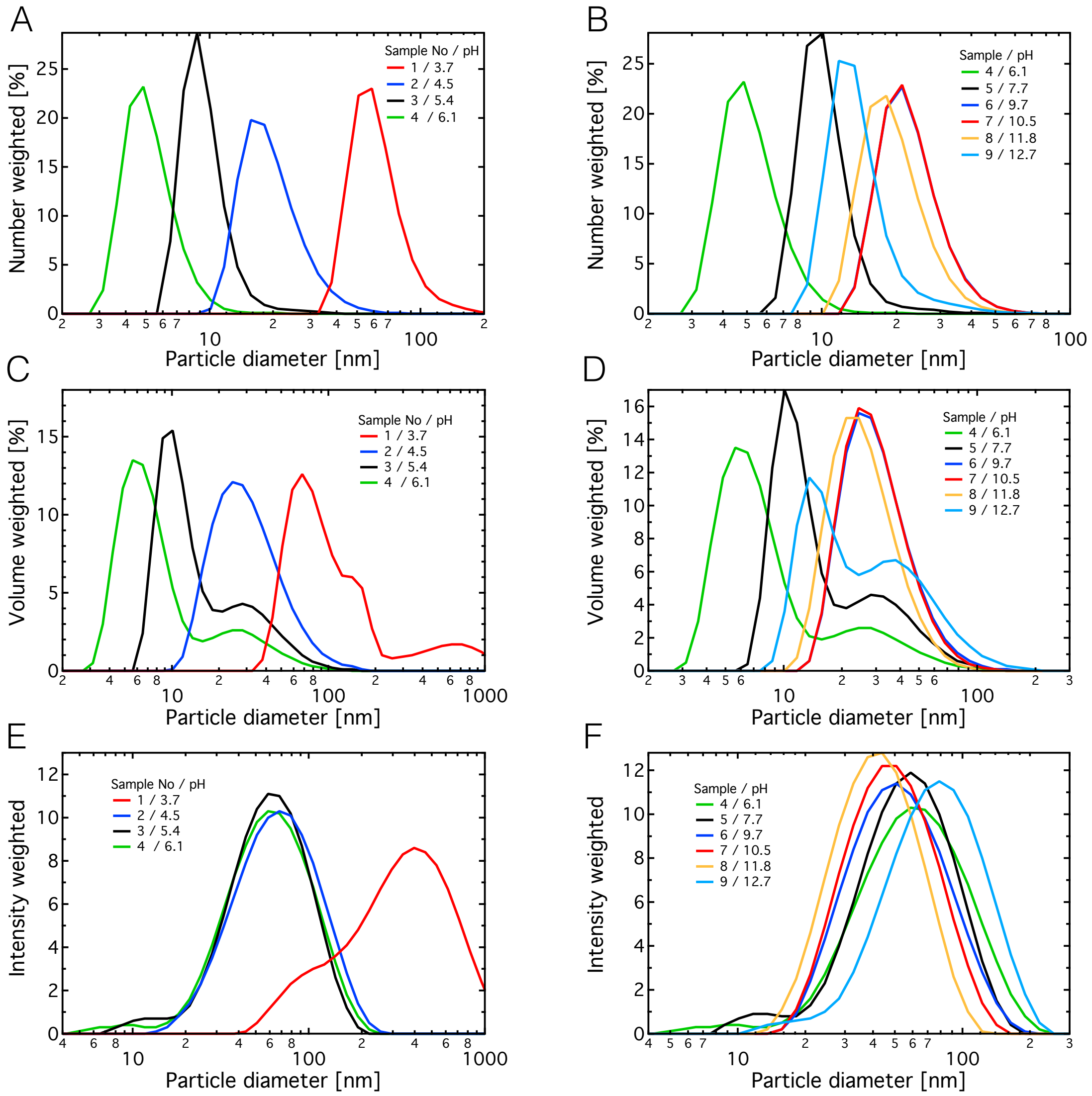

Figure S2. Number weighted (A,B), volume weighted (C,D) and intensity weighted (E,F) particle size distributions as determined by dynamic light scattering for samples 1-4 (A,C,E) and 4-9 (B,D,F) shown in the table above. Measured pH values are indicated in the legend. All size distributions were obtained ~1 minutes after addition of HCl / NaOH as shown in Table 1.

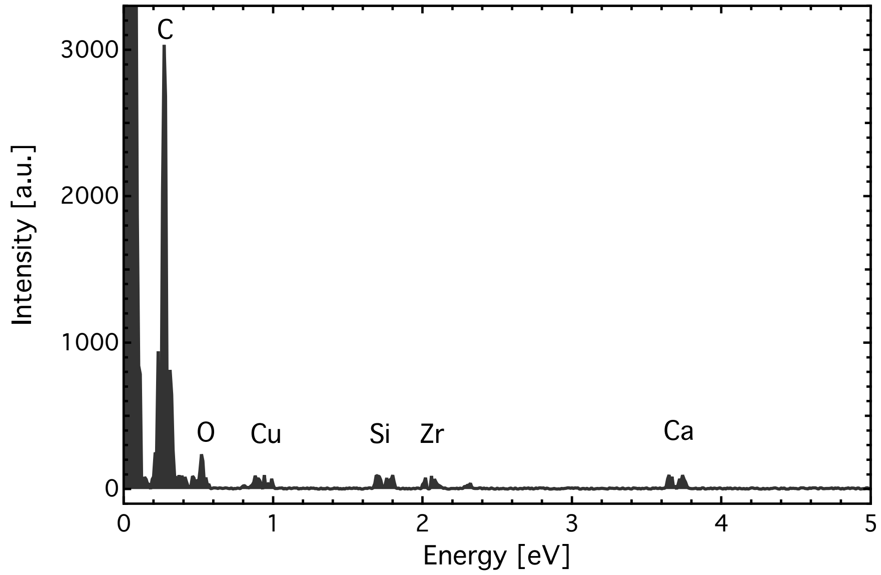

Figure S3. Energy-dispersive X-ray spectroscopy (EDS) results for DND particles show carbon (Kα at 0.277 keV) to be the predominant element in our sample. It also contains significant amounts of oxygen (Kα at 0.525 keV) and we find trace amounts of Cu, Si, Zr and Ca. Particles were deposited on a holey carbon TEM grid.

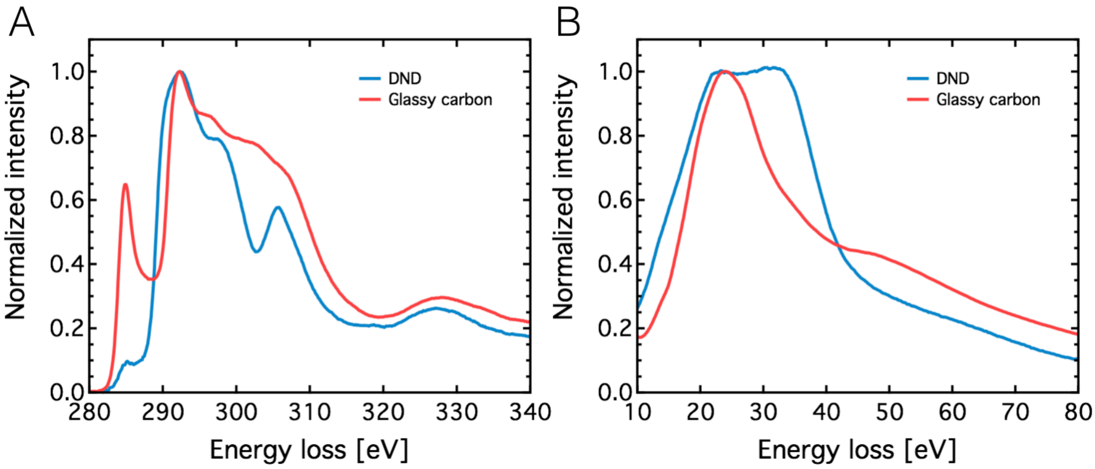

Figure S4. Electron energy loss spectroscopy (EELS) results for the DND particles compared to glassy carbon (GC) used to determine sp^2 and sp^3 carbon content in our samples. A: The low loss EELS spectra for DND and GC. The dominant feature is the plasmon peak which is be a measure of the effective density of the material, assuming a free elctron gas model. For example, GC has a plasmon peak at 22.5eV which equates to a density of 1.54 g cm^{-3}. The plasmon peak of DND however consists of two distinct components with peaks at 22.3 eV (1.51 g cm^{-3}) and 34 eV (3.51 g cm^{-3}). This suggests the presence of diamond as well as graphitic material in our sample. B: Ionization K-edge spectra of the same samples. The presence of both sp^2 and sp^3 bonded carbon is also evident in the ionisiation K-edge spectra where the π* transition is minimal for DND compared to GC and shows that about 18% of the carbon bonds in our sample are sp^2 hybridised and 82 % sp^3 bonded as calculated below.

Acquisition and analysis of EELS spectra

Electron Energy Loss Spectra (EELS) were collected on a JEOL 2100F TEM operating at 200keV with a Gatan Imaging Filter (GIF Tridium) in imaging mode. The Carbon K-edge and low-loss plasmon spectra were both acquired. The K-edge spectra were processed by removing the inherent background and the contribution due to multiple scattering removed. To obtain the sp^2 fraction, the 1s-2π* feature was fitted using a Gaussian distribution and the intensity was compared to the intensity of (1s-2π*) + (1s-2σ). This ratio was compared to the K-edge spectra collected from a glassy carbon sample which is 100% sp^2 according to the below formula

$$sp^2(\%) = \frac{I_{s\pi^*}}{I_{g\pi^*}} \times \frac{I_g(\Delta E)}{I_s(\Delta E)} \times 100$$

Where $I_{s\pi^*}$ is the integral under the 1s-2π* feature of the sample,
$I_s(\Delta E)$ is the integral under the (1s-2π*) + (1s-2σ) features of the sample
$I_{g\pi^*}$ is the integral under 1s-2π* feature of glassy carbon
$I_g(\Delta E)$ is the integral under the (1s-2π*) + (1s-2σ) features of glassy carbon

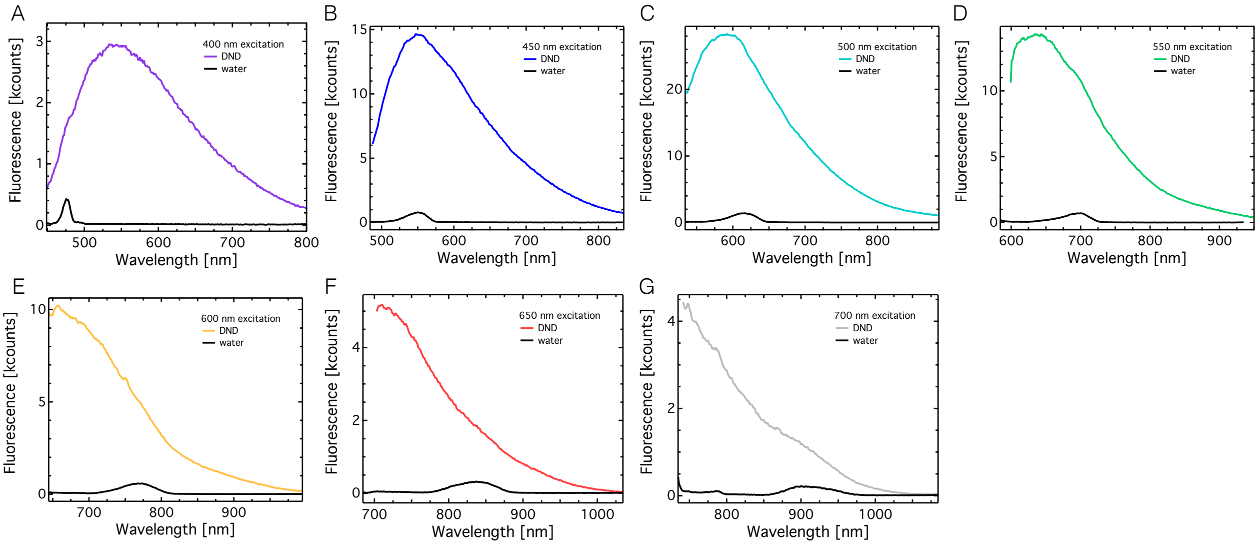

Figure S5. Raw fluorescence spectra for DND samples in water at neutral pH (colored lines) compared to water only (black line) for all excitation wavelengths as indicated in the graphs.

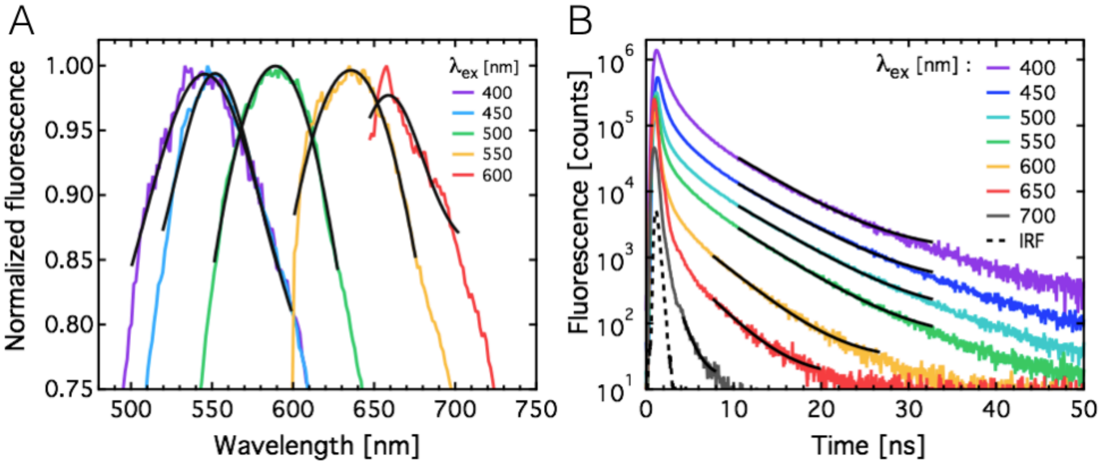

Figure S6. A: Normalized fluorescence spectra for different excitation wavelengths as indicated in the graph. The spectral fluorescence peak position were determined by fitting a the Gaussian

$$f(x) = ae^{-\frac{(x-b)^2}{2c^2}}$$

to the peak of each spectrum (black lines), where the fitting parameter 'b' was used as the values λ_{em} for plotted in Figure 3B in the main text.
B: Fluorescence decay traces for the different excitation wavelengths as indicated in the graph. The long fluorescence lifetime component Tau2 was determined by fitting a single exponential to the decay traces a shown in the graph (black lines). We find the lifetimes determined this way to vary by ±0.35 ns depending on the exact region used for fitting, which is reflected in the error bars shown in Figure 3C in the main text. The same approach was used for the analysis of the pH dependent results shown in Figure 5 on the main text.

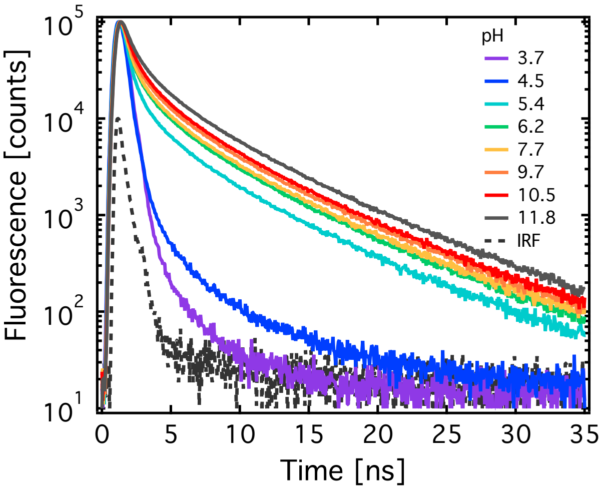

Figure S7. Time resolved fluorescence decay traces for all pH values investigated except 12.7, which is omitted for clarity.

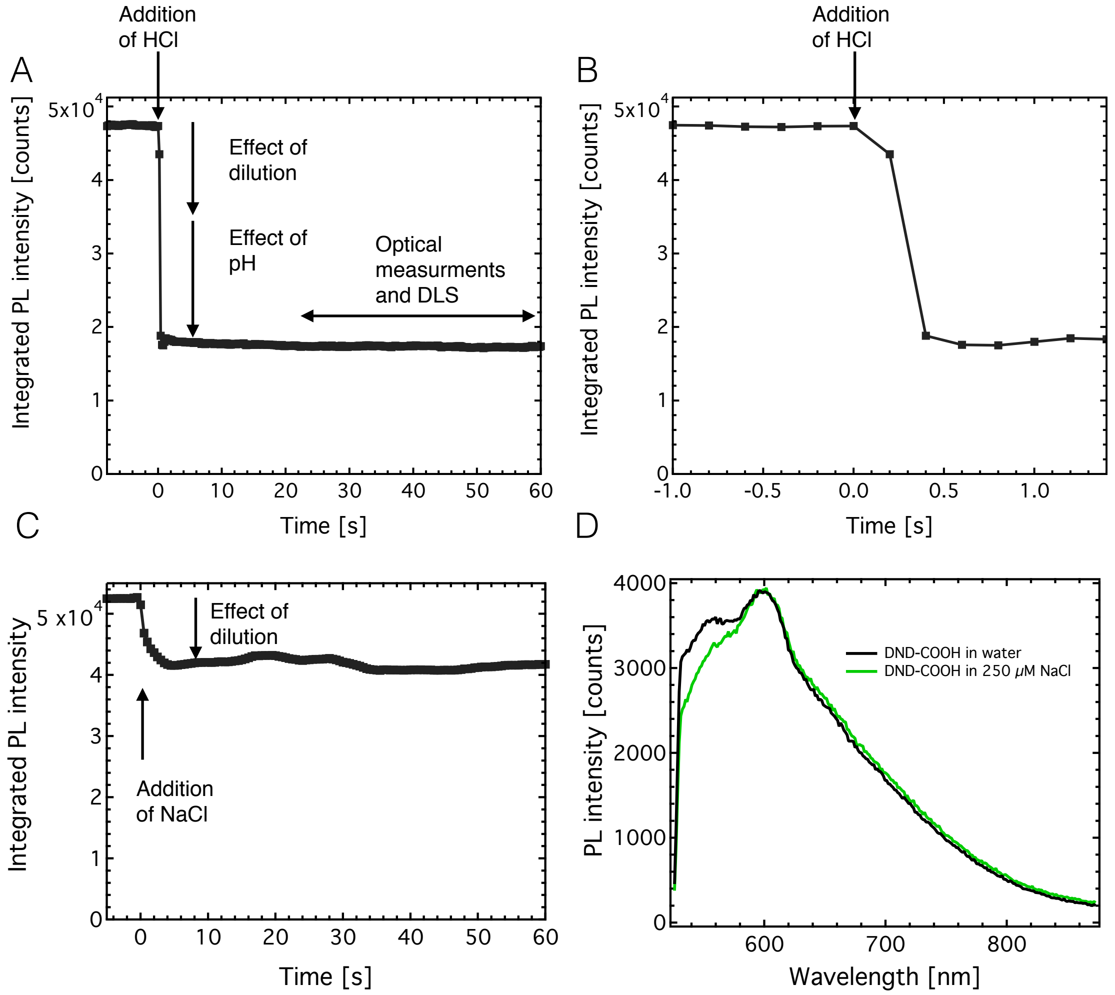

Figure S8. A: Integrated fluorescence intensity as a function of time. A DND nanoparticle solution (300 µL, 1.33 mg/mL) was excited with 500 nm light and the fluorescence collected with a spectrometer at 5 frames per second. An aqueous solution of HCl (100 µL, 1 mM) was added at time t=0 seconds. The fluorescence decreases by 75% within less than 0.5 s and remains stable thereafter. This decrease is caused by a dilution of the starting solution by 33% as well as the decrease in pH, resulting in a decreases of fluorescence in agreement with Figure 5B in the main text. Nanoparticle aggregation is a diffusion-limited process. The mean displacement of a 5 nm spherical particle due to Brownian motion after 500 ms is below 1 nm, which makes a collision with another particle (as a prerequisite for aggregation to occur) within this timeframe highly improbable in our nanoparticle solutions. B: Same data as in A, but zoomed into the region where the HCl addition occurs. C: The same experiment as in A, but using NaCL instead of HCl. Here, the intensity decreases due to the dilution of the solution, but only by around 23% instead of the expected 33% dilution. This is likely caused by incomplete mixing, which can be difficult to achieve in these measurements. D: Fluorescence spectra of DND particles dispersed in water (black line) and in 250 µM NaCl (green line), which is the final HCl concentration used in panel A and NaCl concentration used in panel C. The spectra were measured ~ 30 s after the addition of either water or NaCl. In the presence of salt the spectrum shows a slight red-shift, which is typical for partially aggregated particles. Overall, the difference in fluorescence intensity is < 1%, which is within the experimental

error. These experiments unambiguously demonstrate that the fluorescence decrease is not caused by aggregation or the presence of salt ions.

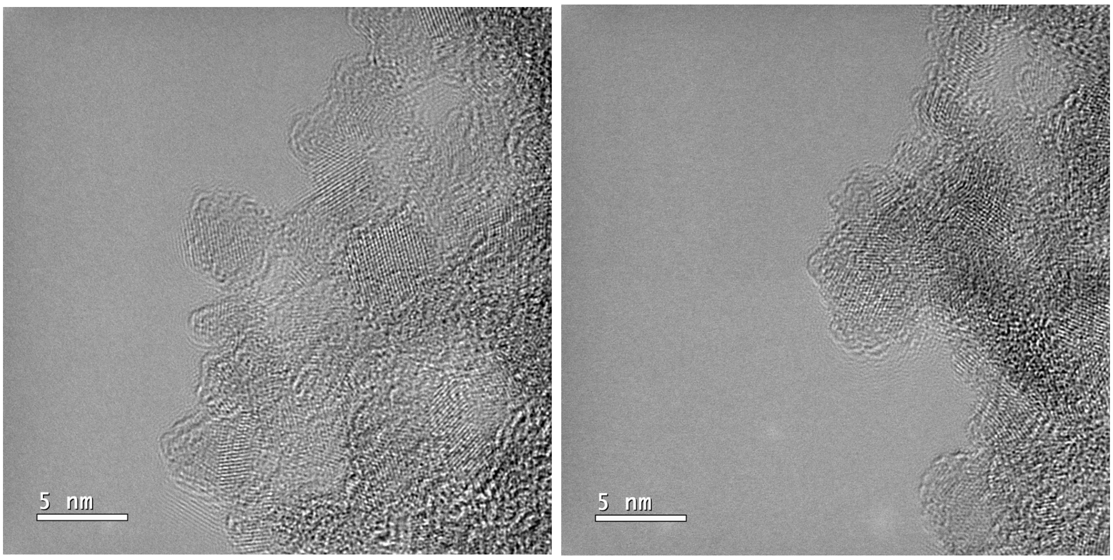

Figure S9. TEM images of the DND particles. Images were acquired with a JEOL 2100F TEM operating at 200keV as described above under 'Acquisition and analysis of EELS spectra'.

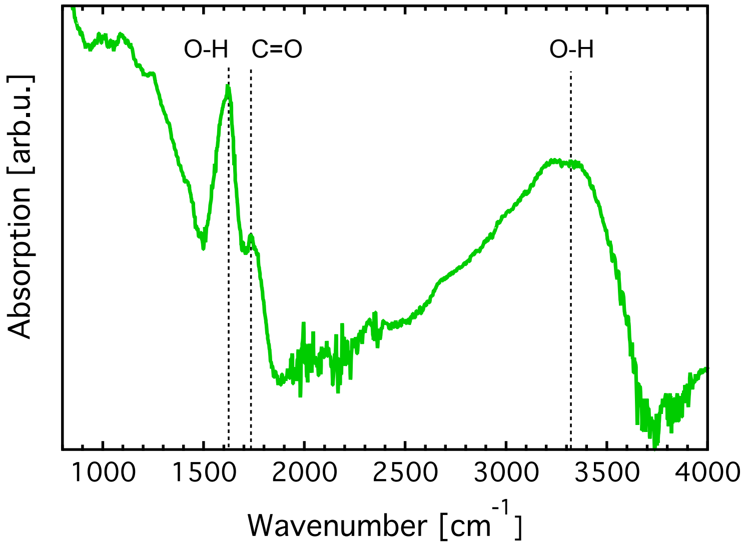

Figure S10. FTIR spectrum of the investigated DND particles. Absorption peak positions of O-H (stretch and bend) and C=O (stretch) vibrations of carboxyl groups are indicated. The spectrum was recorded using a Perkin Elmer Frontier FTIR spectrometer fitted with an ATR attachment. Nanoparticle powder was obtained via drying of the nanoparticle solution.

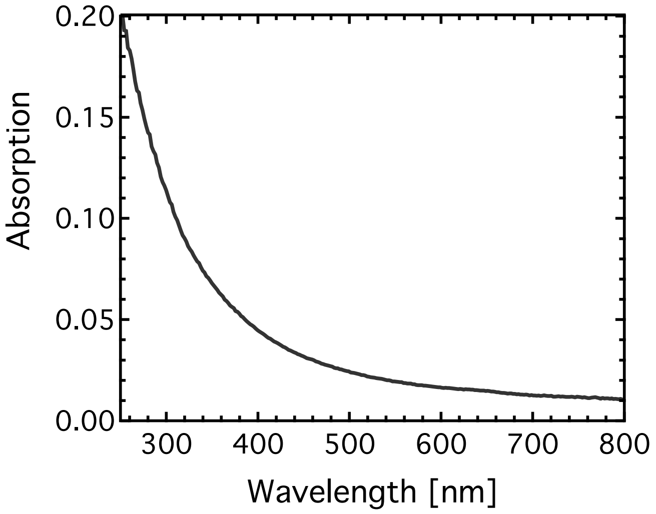

Figure S11. Absorption spectrum of DND particles in water (0.04 mg/ mL) for the spectral range from 250 nm to 800 nm. The data was acquired using a Cary 700 absorption spectrometer (Agilent Technologies) and an integrating sphere.

Calculation of the Debye length

The Debye length λ_D is commonly defined by the equation[1]:

$$\lambda_D = \sqrt{\frac{\varepsilon_r \varepsilon_0 k_B T}{\sum_{i=0}^{N} c_i e^2 z_i^2}}$$

where ε_0 is the permittivity of free space, ε_r the dielectric constant of the solvent, $k_B T$ the thermal energy, c_i the ionic concentration of the i-th ion species in solution, e the elementary charge and z_i the valency of the i-th ion species. The Debye length is a characteristic length for the range of the electrostatic potential into the solvent. For monovalent ions and a molar electrolyte concentration [C] it simplifies to (expressed in nanometers):

$$\lambda_D = \frac{0.304}{\sqrt{[C]}} nm$$

For $[Na^+] \approx 0.005$ M this yields 4.3 nm compared to 961 nm for $[C] \approx 0.0000001$ M ($[H^+]$ in water) at neutral pH.

Calculation of mean square displacement

The mean square displacement X of a particle in 3 dimenasions was estimated using the equation

$$X = 3Dt$$

where D is the Stokes-Einstein diffusion coefficient and t is time. D was calculated using the following equation and parameters:

$$D = \frac{k_B T}{6\,\pi\,\eta r}$$

$k_B T$ = 4.11 E-21 J (thermal energy)
η = 8.94 E-04 kg m^{-1} s^{-1} (viscosity of water)
r = 2.5 nm (particle radius)

For a diffusion time of 1 second and a particle size of 5 nm (most particles are larger than this so this is an upper bound for X) the mean square displacement is ~0.3 nm.

Estimation of the average particle separation in solution

We have used the Wigner–Seitz radius to estimate the average separation of DND particles dispersed in water at a concentration of 3 µM or ~ 1.8 e18 particles per liter (1 dm^3). The Wigner–Seitz radius in 3D is given by:

$$R_s = \sqrt[3]{\frac{3V}{4\pi N}}$$

where V is the volume of the solvent and N the number of particles. Using the values given above one obtains R_s= 51 nm. The nearest neighbor distance would thus be 102 nm (2 R_s). This value is more than two orders of magnitude higher than the mean square displacement of 5 nm particles after diffusing for 1 second in water.

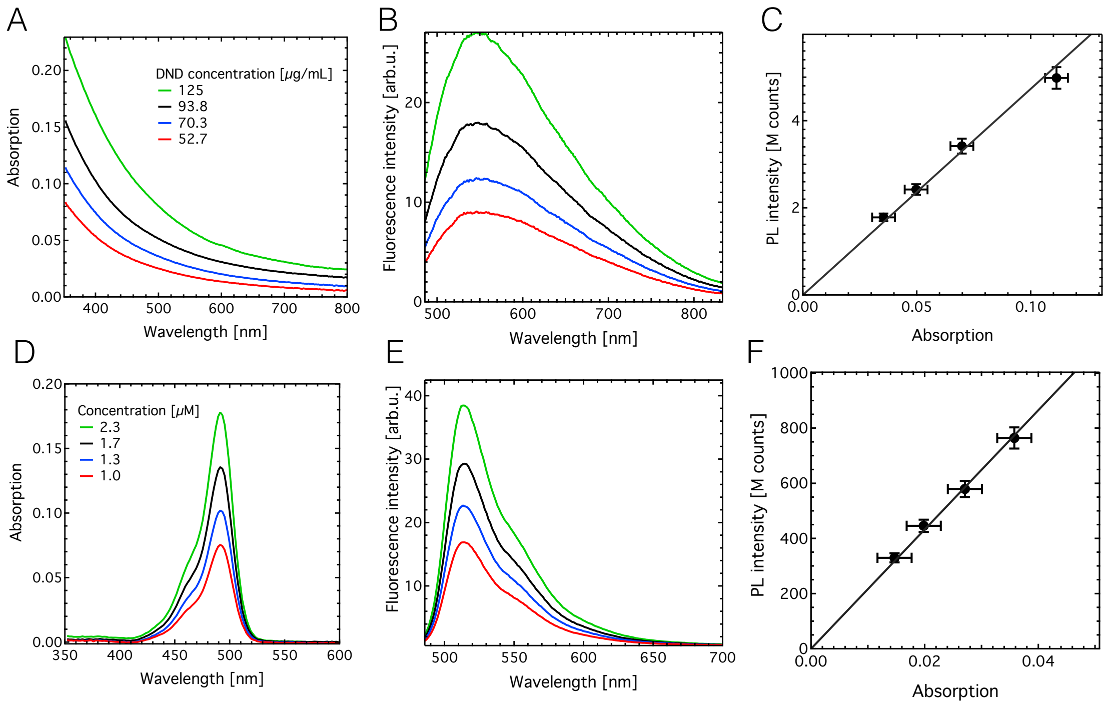

Figure S12. Determination of the fluorescence quantum yield for 450 nm excitation relative to fluorescein in 10 mM NaOH. A,D: absorption spectra; B,E: fluorescence spectra; C,F: Integrated fluorescence intensity as a function of absorption.

Determination of the relative fluorescence quantum yield

The quantum yield (Φ) was determined using the equation:

$$\Phi_{DND} = \Phi_F \times \frac{b_{DND}}{b_{FITC}}$$

where Φ_{DND} and Φ_F are the quantum yields of DND and fluorescein ,respectively, and the corresponding gradients b of the fits to the data shown in Figure S12 C and F using the equation $y = b\,x$. The quantum yield of fluorescein in 10 mM NaOH of Φ_F=0.93 was used as reported by Kubista et al.[2] This yields a value of Φ_{DND} = 0.22% for the quantum yield of the DND particles.

References

1. Hunter, R. J. *Foundations of colloid science / Robert J. Hunter*. (Oxford University Press Oxford ; New York, 2001).
2. Sjöback, R., Nygren, J. & Kubista, M. Absorption and fluorescence properties of fluorescein. *Spectrochim. Acta Part A Mol. Biomol. Spectrosc.* **51,** L7--L21 (1995).
